# Patient fibroblast circadian rhythms predict lithium sensitivity in bipolar disorder

**DOI:** 10.1038/s41380-020-0769-6

**Published:** 2020-05-13

**Authors:** Harshmeena R. Sanghani, Aarti Jagannath, Thomas Humberstone, Farid Ebrahimjee, Justyn M. Thomas, Grant C. Churchill, Andrea Cipriani, Mary-Jane Attenburrow, Olga V. Perestenko, Sally A. Cowley, M. Zameel Cader, Stuart N. Peirson, Paul J. Harrison, Russell G. Foster, Guy M. Goodwin, Sridhar R. Vasudevan

**Affiliations:** 1 Department of Pharmacology, University of Oxford, Mansfield Road, Oxford, OX1 3QT UK; 2 Nuffield Department of Clinical Neurosciences, University of Oxford, OMPI-G, South Parks Road, Oxford, OX1 3RE UK; 3 Addenbrookes Hospital, Cambridge University Hospitals NHS Foundation Trust, Cambridge, CB2 0QH UK; 4 Department of Psychiatry, Warneford Hospital, University of Oxford, Oxford, OX3 7JX UK; 5 Oxford Health NHS Foundation Trust, Warneford Hospital, Oxford, OX3 7JX UK; 6 Sir William Dunn School of Pathology, University of Oxford, South Parks Road, Oxford, OX1 3RE UK; 7 Weatherall Institute of Molecular Medicine, University of Oxford, Oxford, OX3 9DS UK

**Keywords:** Bipolar disorder, Drug discovery

## Abstract

Bipolar disorder is a chronic neuropsychiatric condition associated with mood instability, where patients present significant sleep and circadian rhythm abnormalities. Currently, the pathophysiology of bipolar disorder remains elusive, but treatment with lithium continues as the benchmark pharmacotherapy, functioning as a potent mood stabilizer in most, but not all patients. Lithium is well documented to induce period lengthening and amplitude enhancement of the circadian clock. Based on this, we sought to investigate whether lithium differentially impacts circadian rhythms in bipolar patient cell lines and crucially if lithium’s effect on the clock is fundamental to its mood-stabilizing effects. We analyzed the circadian rhythms of bipolar patient-derived fibroblasts (*n* = 39) and their responses to lithium and three further chronomodulators. Here we show, relative to controls (*n* = 23), patients exhibited a wider distribution of circadian period (*p* < 0.05), and that patients with longer periods were medicated with a wider range of drugs, suggesting lower effectiveness of lithium. In agreement, patient fibroblasts with longer periods displayed muted circadian responses to lithium as well as to other chronomodulators that phenocopy lithium. These results show that lithium differentially impacts the circadian system in a patient-specific manner and its effect is dependent on the patient’s circadian phenotype. We also found that lithium-induced behavioral changes in mice were phenocopied by modulation of the circadian system with drugs that target the clock, and that a dysfunctional clock ablates this response. Thus, chronomodulatory compounds offer a promising route to a novel treatment paradigm. These findings, upon larger-scale validation, could facilitate the implementation of a personalized approach for mood stabilization.

## Introduction

Bipolar disorder (BD) is a chronic illness characterized by recurrent episodes of abnormal mood. It affects 1–3% of the population worldwide and is one of the major causes of chronic disability [1, 2]. The first-line treatment is lithium, functioning as a potent mood stabilizer in most patients [3–5]. Little is known about the molecular pathways that cause BD, other than hypotheses that build on a range of targets of lithium [6]. As a consequence, BD has been difficult to model in vitro, thus hampering drug discovery. One leading hypothesis is that lithium modulates circadian gene expression, and thus the stabilization of sleep and circadian rhythms might offer novel treatment paradigms.

Sleep and circadian rhythm disruption have been noted in many neuropsychiatric disorders, including BD [7–9]. Virtually all aspects of mammalian physiology and behavior display 24 h circadian rhythms, driven by a series of transcriptional–translational feedback loops that yield cell-autonomous rhythm generation. The core of this molecular clock is composed of CLOCK and BMAL1, which regulate the expression of their negative repressors *Per (Period 1/2/3)* and *Cry (Cryptochrome 1/2)*, thus forming a process that repeats every 24 h [10]. Indeed, the comorbid nature of sleep/circadian disruption and mood is now well established [11–13]: Diagnostic and Statistical Manual of Mental Disorders 5 now recognizes this as a diagnostic criterion for manic and depressive episodes [14].

At the mechanistic level, genes such as *Clock, Bmal1*, and *Per*, which are intimately involved in the generation and regulation of circadian rhythms, have been linked to BD [15, 16], and this is supported by mouse models. *ClockΔ19* mutant mice, for example, in addition to displaying sleep and circadian deficits (reduced sleep requirement and increased circadian period), display mania-like behaviors including reduced anxiety and depression-like behaviors, as well as increased risk taking and hyperhedonia [17, 18]. Consistent with the *ClockΔ19* mutant, *Afh* (*After-hours*) mice which carry a mutation in the clock-gene *Fbxl3* thereby preventing *Cry (1/2)* degradation, display significantly altered rhythms and a mania-like phenotype [19–21], further reinforcing a strong link between period length and mood-related behaviors, and by extension BD and clock genes.

The highly accessible nature of skin cells and their ability to pass on circadian timing to daughter cells, make fibroblasts a valid and promising tool for assessing human circadian rhythmicity [22]. Previous use of healthy human or murine cells have found that the molecular clocks within fibroblasts provide an accurate indication of rhythms generated in vivo and by the SCN [23–25]. Previous clock-gene luminometric analysis found the period to be elongated by up to 25 min in BD patient cells [26]. A subsequent study found that BD fibroblasts exhibited nonsignificant (40 min) trends in period lengthening [27] (Table 1).

**Table 1:** Summary of previous fibroblast bioluminescence reporter studies assessing molecular rhythmicity from healthy controls or bipolar patients.

Subject	*n*	Gene	Period (h)	Publication
Control	19	*Bmal1*	24.5 ± 0.75 (s.d.)	Brown et al. [23]
Control	28	*Bmal1*	24.5 ± 0.75 (s.e.m.)	Brown et al. [25]
	9		24.71 ± 0.38 (s.e.m.)	Pagani et al. [24]
Control	11	*Bmal1*	24.77 ± 0.42 (s.e.m.)	Pagani et al. [24]
	8		24.46 ± 0.48 (s.e.m.)	Pagani et al. [24]
Control	19	*Per2*	25.10 ± 0.20 (s.e.m.)	McCarthy et al. [26]
BD	19		25.50 ± 0.20 (s.e.m.)^a^	McCarthy et al. [26]
Control	12	*Bmal1*	25.50 ± 0.60 (s.d.)	Bamne et al. [27]
BD	13		26.30 ± 1.20 (s.d.)	Bamne et al. [27]

^a^Indicates a significant difference.

Alongside these circadian abnormalities in BD, several mood stabilizers, including lithium, have an effect on the clock [28–30]. Lithium has been shown to increase the amplitude and period of circadian rhythms in a range of organisms from plants to humans [31–34]. As the targets of lithium are many and various downstream consequences remain unknown, the mechanism by which lithium acts on the circadian system may be an attractive target for mood modulation. Further, recent evidence of lithium’s action on the clock comes from effects on BD patient lymphoblastic cell lines [35]. Two days after treatment, cells from lithium-responsive patients (unlike those from nonresponsive patients) demonstrated elevated expression of the core clock components *Bmal1, Per1*, and *Cry2*, providing further clinical insight into the effects of lithium.

Here we have analyzed the circadian rhythms and chronomodulatory drug responses of BD patient-derived fibroblasts. We sought to answer three questions: (1) does lithium differentially impact circadian rhythms in different patient cell lines; (2) is lithium’s effect on the clock necessary for mood stabilization; and (3) what factors predispose individuals toward sensitivity to lithium. While lithium remains the first-line drug for BD, clinical response varies. Accordingly, polypharmacy is common, and often therapeutically necessary. We hypothesized that patients taking fewer medications do so because their circadian clocks respond well to lithium treatment, whereas those that need a greater number of medications have a circadian clock that is less responsive to chronomodulation.

We found that BD patients exhibited a wide distribution of period length. Those with longer basal circadian periods took more medications overall and displayed attenuated responses to lithium (as well as alternative validated chronomodulators), implying that lithium differentially impacts the circadian system and that efficacy is determined by the patients underlying circadian phenotype. Furthermore, we show that drugs that phenocopy lithium’s circadian response in patient fibroblasts elicit behavioral alterations akin to those for lithium in mice, and that a dysfunctional clock grossly alters murine lithium-induced behavioral responses, suggesting an intricate link between the circadian clock and lithium therapy.

## Patients and methods

### Compounds and reagents

All reagents were from Sigma-Aldrich except where stated otherwise. Nuclease-free water or sterile DMSO was used to prepare the compounds.

### Subject characteristics

The control subjects who donated fibroblasts had an average age of 53.56 ± 3.18 years. The BD subjects had an average age of 43.10 ± 1.97 years; 82% were diagnosed with BP-I disorder, and 67% presented with psychotic symptoms. The patients were further divided into those who were lithium-treated (Li-T) and lithium-non-treated (Li-NT); 77% of the patients were taking lithium at the time of the biopsy (Table S1) for an average of 10 years (Table S2). Only 20% of patients were on lithium alone. In the rest, an assortment of additional drugs (up to six) had been added to control mood instability. The drugs were divided into five classes by indication (Tables S3, S4). Patients were taking on average a combination of two or three compounds across multiple drug classes; mood stabilizers and antipsychotics were the medications most frequently reported.

### Cell lines

BD patient-derived dermal fibroblasts (*n* = 39) and control subject-derived dermal fibroblasts (*n* = 23) were obtained from the StemBANCC consortia, who oversaw the collection and culturing of cells from 500 individuals under standardized conditions. Fibroblasts were derived from 3 mm punch-skin biopsies, following informed consent in accordance with StemBANCC ethics practice [36]. Biopsies were plated in Advanced DMEM (ADMEM, ThermoFisher Scientific, Waltham, Massachusetts, USA) with 10% Fetal Bovine Serum (FBS) for outgrowth of fibroblasts, with subsequent expansion for generating a banked frozen stock in 10% DMSO at passage 2 in nitrogen vapor. This was further expanded for functional studies, which were carried out at passages 4–6.

### Lentivirus production and cell-line generation

Mass production of *Bmal1*-Luc or *Per2*-Luc virus was performed to infect fibroblasts with the same batch of lentivirus. HEK-293 cells were seeded in HYPERflasks (Corning, Corning, New York, USA). The plasmid complex included: 60.4 μg reporter plasmid; *Per2* (gift from Qing-Jung Meng Lab) [37] and *Bmal1* (pABpuro-BluF was a gift from Steven Brown (Addgene plasmid # 46824)), 40.25 μg packaging (psPAX2 was a gift from Didier Trono Addgene plasmid # 12260), 16.1 μg envelope plasmid (pMD2.G was a gift from Didier Trono (Addgene plasmid # 12259), and 1 mL of PEI-25 kDa (1 mg/mL; Polyscience, Warrington, Pennsylvania, USA) as the transfection reagent. Then, 16 h post transfection, the cellular media (DMEM, Sigma-Aldrich, St. Louis, Missouri, USA) was replaced with fresh media and incubated at 37 °C for 48 h, as previously described [38]. The viral supernatant was concentrated in a Vivaspin 20 and quantified using a p24 ELISA (Takara Bio, Paris, France).

Fibroblasts grown in 75 cm^2^ flasks were transduced with a spinfection protocol. Briefly, 100,000 fibroblast cells to be transduced were lifted with TrypLE (ThermoScientific) and centrifuged with 200 µl virus (~MOI 20) at 3000 × *g*, 25 °C for 99 min. Cells were then resuspended in viral medium and incubated at 37 °C overnight prior to use.

### Assessing circadian rhythms

Fibroblasts were cultured to confluence (~30,000 cells/well) in 96-well white plates (Greiner Bio-One, Gloucestershire, UK) using ADMEM (ThermoScientific) containing 10% FBS and 1% penicillin–streptomycin. Cells were synchronized with 200 nM dexamethasone, diluted in serum-free medium for an hour and washed twice with the same solution (ThermoScientific) before reconstituting with serum-free medium containing 1× B27 (ThermoScientific) and 400 μM luciferin (Gold Biotechnology, St. Louis, Missouri, USA). Bioluminescence was recorded for 4 days in Tecan M200 Pro readers. Actimetrics MultiCycle was used to determine the period and amplitude (baseline subtracted 24 h running average of the raw luminescence data smoothed over 8 h). For luminometric experiments involving the 62 fibroblast cell lines, three technical replicates per dose, per drug, were conducted once and averaged.

### Correlograms

**Fig. 1:**
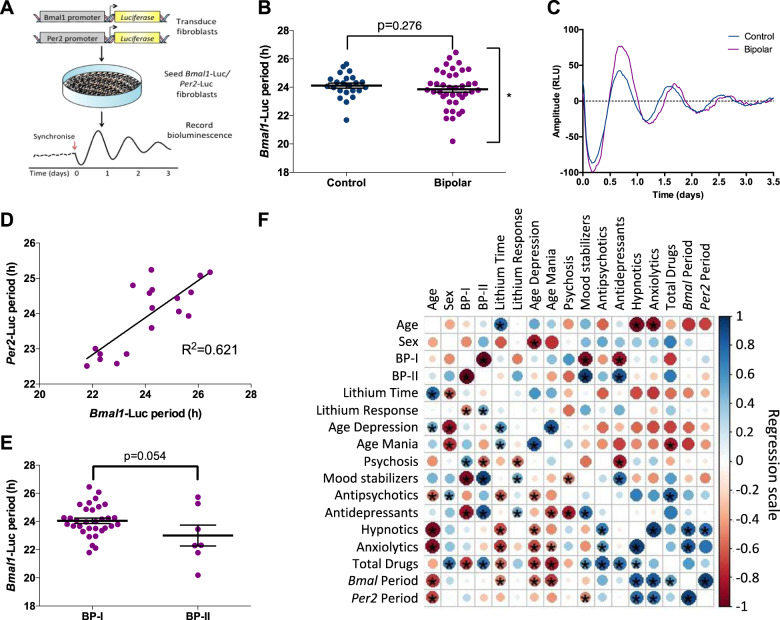
Lentivirus-transduced BP patient fibroblasts express a variable underlying circadian phenotype. **a** Protocol to assess circadian rhythmicity. **b** *Bmal1*-Luc period differences between control (*n* = 23) or BD patient (*n* = 39) fibroblasts were analyzed using two-tailed Student’s *t* test with Welch’s correction, enabling variance analysis. **c** Example oscillations from control and BD cells transduced with the *Bmal1*-Luc lentivirus. **d** Linear regression comparing *Bmal1*-Luc rhythms with *Per2*-Luc rhythms in BD patient-derived fibroblasts (*n* = 18) reveals a correlation (*R*^2^ = 0.621). **e** Comparison between BP-I and BP-II cellular rhythms. **f** Correlogram revealing correlative patterns and their significance using a Pearson parametric correlation test on a scale of +1 to −1. Blue indicates positive correlation; red for negative correlation; larger circles represent stronger correlation. Results from a Student’s *t* test are presented in the bottom left triangle, where the top right indicates significance following the Holm–Bonferroni test. Data are mean ± s.e.m. *p < 0.05.

The statistical program R generated correlograms which portray the correlation coefficient using a Pearson’s parametric correlation test from +1 to −1 in Fig. 1f. Blue and red are indicative of a positive and negative correlation respectively; a larger circle represents stronger correlation. Significance was assessed using a two-tailed *t* test (bottom left); top right indicates significance following adjustments for multiple comparisons using the Holm–Bonferroni test.

### Animals

Wild-type male C57BL/6J (Envigo, Huntingdon, UK) and male *Cry1/2*^*−/*−^ mice (a kind gift from Patrick Nolan, MRC, Harwell, UK) of 8–12 weeks of age were housed under 12 h:12 h LD cycles for 1 week. The *Cry1/2*^−*/*−^ mice [39] were maintained as a homozygous line in the C57BL/6J background with regular backcrossing. Animals were housed and assessed in a randomized manner across treatment groups to avoid effects associated with housing location or order of treatments. All analyses were automated to remove user involvement. Each experiment involving a particular treatment group/genotype was performed on a cohort of the indicated size on a single occasion. Sample sizes were estimated based on evidence from publications and past experiments within our laboratory. Wild-type mice were injected intraperitoneally (i.p.) with either vehicle (*n* = 7), 200 mg/kg LiCl (*n* = 6), 50 mg/kg nobiletin (NOB) (*n* = 7), or 5 mg/kg CGS-15943 (CGS) (*n* = 7). An independent experiment assessing the impact of 200 mg/kg LiCl on *Cry1/2*^−*/*−^ mice was also conducted (vehicle^*+/+*^: *n* = 6, LiCl^*+/+*^: *n* = 7, vehicle^*Cry1/2*−/−^: *n* = 8, LiCl^*Cry1/2*−/−^: *n* = 8). One mouse from the wild-type LiCl group and one mouse from the vehicle group were excluded due to in-cage fighting. The drugs were diluted in 0.9% NaCl with 5% kolliphor HS 15 and 5% 2-hydroxypropyl-β-cyclodextrin, except for LiCl, which was diluted in 0.9% NaCl. Compounds were administered for 10–12 days. The lithium dosage (200 mg/kg) was chosen based on previous studies [40]. All procedures were performed in accordance with the UK Home Office Animals (Scientific Procedures) Act 1986 and the University of Oxford’s and NC3R’s policy on the Use of Animals in Scientific Research.

### Open field test

The open field test (OFT) was performed using the Linton AM1053 X, Y, Z IR Activity Monitor (San Diego Instruments, San Diego, California, USA). Animals were assessed in clear Perspex cages (36 × 20 × 15 cm) consisting of two parallel frames of 24 equally spaced beams along the *X* and *Y* (8 × 16) axis. Animal activity was scored in 1 min time bins for 20 min.

### Elevated plus maze

The elevated plus maze (EPM) utilized a maze elevated 47 cm off the ground. The apparatus consisted of a central platform (5 × 5 cm) with two opposing open arms (28 × 5 cm) lit to 290 lux and two opposing close arms (28 × 5 × 30 cm, 50 lux). Each mouse was placed on the central platform facing the closed arms and animal activity was recorded for 10 min. Video analysis was performed using ANY-maze 5.3 (Stoelting Co., Wood Dale, Illinois, USA). Mice that fell or jumped off the EPM apparatus were excluded from further analysis as these experiments were performed without the user being present in the room.

### Forced swim (Porsolt) test

The forced swim test (FST) was performed in a glass cylinder (18 cm diameter, 27 cm height) filled with 3.5 L water (24 ± 1 °C), a depth at which the animal’s tail is unable to touch the bottom of the apparatus. Each mouse was kept in the water for 6 min before being dried in a clean cage and returned to the home cage. The water was changed after each subject. Video analysis was performed using ANY-maze 5.3.

### Data analysis

Statistical analysis was performed using GraphPad Prism; the results are presented as average ± s.e.m. The threshold for significance was *p* < 0.05. An unpaired two-tailed *t* test or welches *t* test was performed when comparing two parameters. A one-way ANOVA with a Tukey or Dunnett’s post hoc test was used when comparing multiple parameters. A two-way ANOVA with a Tukey or Sidak’s post hoc test was applied when two independent variables were present.

## Results

### The underlying circadian phenotype in BD

In order to perform luminometric recordings and analysis of circadian rhythms in vitro, fibroblasts from BD patients and controls were transduced with lentivirus where luciferase expression was driven by activity at the *Bmal1* or *Per2* promoter regions (Fig. 1a). Following the creation of stable cell lines, the fibroblasts were synchronized with 200 nM dexamethasone, and basal period length was analyzed. Both BD and control cells exhibited a normally distributed period, but the BD cohort presented with a larger distribution and significantly greater variance, detected using the Welch’s correction (control period: 21.69–25.64 h; BD: 20.19–26.45 h; *p* = 0.039) (Fig. 1b). We saw no difference in basal period in BD patients compared with the controls (control: 24.03 ± 0.20 h, BD: 23.87 ± 0.21 h) (Fig. 1b, c). To validate this observation, an additional study with *Per2* gene expression was performed on a cellular subset (Fig. S1A, B), and similar observations were obtained (control: 23.48 ± 0.27 h, bipolar: 23.93 ± 0.23 h). Regression analysis to assess the correlation between *Bmal1* and *Per2* bioluminescence rhythms (Fig. 1d) yielded a positive correlation (*R*^2^ = 0.621); patients with longer *Bmal1* molecular rhythms were more likely to display longer *Per2* rhythms.

We explored whether known demographic characteristics were responsible for the period variability within our BD subject cell lines. BD and control cells displayed similar growth rates (Fig. S1C). Furthermore, sex, age, the presence of psychotic symptoms, and other key features analyzed presented with no significant basal differences (Fig. S1D–H). Interestingly, the emergent trend (Fig. 1e) suggested that patients diagnosed with bipolar II disorder (BP-II—who experience hypomania, and depression is more prominent) had a period that was on average 1.1 h shorter than those with bipolar I disorder (BP-I—mania is more prominent) (BP-I: 24.05 ± 0.19, BP-II: 23.00 ± 0.74 h; *p* = 0.054) [14]. This was the only parameter that approached significance in accounting for the variability observed (Fig. 1b).

We next explored the emergent correlations between circadian rhythms and treatment regime for the BD data in the *Per2* and *Bmal1* regression analysis in Fig. 1d, f. We detected a relationship between periodicity and medication: patients with a shorter circadian period were taking more mood stabilizers. By contrast, patients with a longer period were taking more hypnotics and anxiolytics. Furthermore, the longer the circadian period, the greater the total number of medications. Thus, this offered preliminary support for our hypothesis that patients exhibiting longer basal periods are less responsive to chronomodulation by lithium.

### Patient stratification reveals circadian drug responses to be a determinant of basal circadian rhythms

**Fig. 2:**
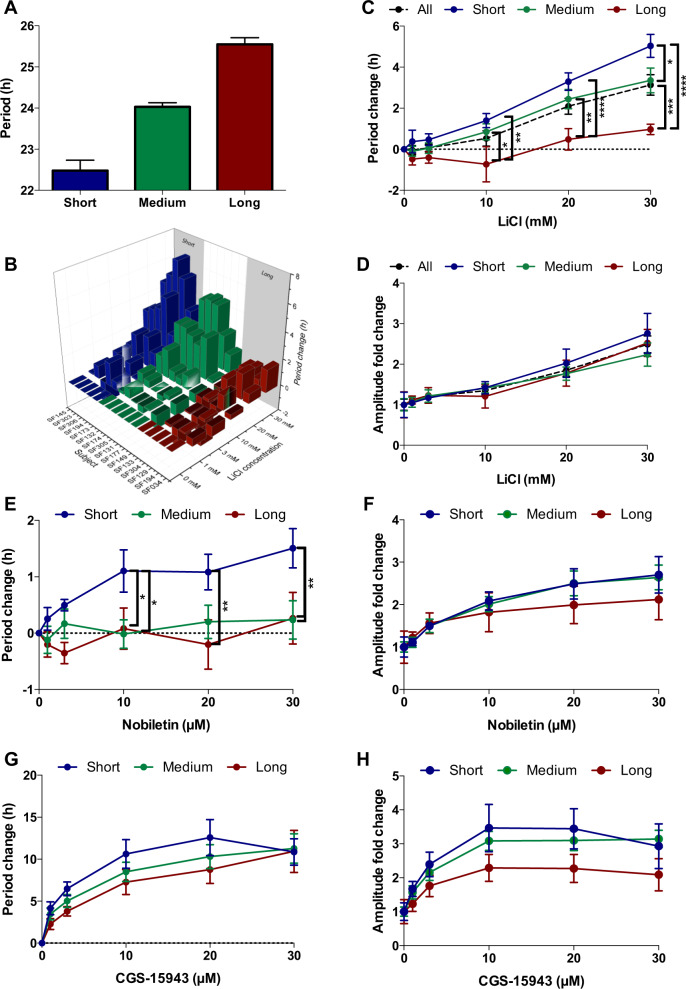
The underlying patient-derived cellular circadian phenotype determines patient chronomodulatory responses to lithium, nobiletin, and CGS-15943. **a** Patients were divided into groups based on their basal *Bmal1*-Luc period length: short (23.49 h or less, *n* = 13), medium (23.50–24.99 h, *n* = 17), and long (25.00 h or more, *n* = 9). **b** 3D plot displaying the variable dose-dependent period change induced by LiCl in 16 different BD patient cell lines. **c** Period and **d** amplitude effects induced by 1–30 mM LiCl in short (*n* = 5), medium (*n* = 6), or long (*n* = 5) BD fibroblasts. **e**, **f** Period and amplitude changes induced by NOB in short (*n* = 5), medium (*n* = 6), or long (*n* = 5) cells. **g**, **h** Chronomodulatory changes induced by CGS in short (*n* = 5), medium (*n* = 6), or long (*n* = 5) fibroblasts. **c**–**h** Data analysis was performed using a two-way ANOVA with a Tukey’s post hoc test. Data are mean ± s.e.m. *p < 0.05; **p < 0.01; ***p < 0.001; ****p < 0.0001.

Our regression analysis (Fig. 1f) highlighted that patients with longer periods were taking more medications. Hence, to test the hypothesis that patients with longer periods are less sensitive to lithium-induced chronomodulation, patients were divided into three categories (Fig. 2a, Fig. S2A) based on period length: short (up to 23.49 h), medium (23.50–24.99 h), and long (25.00 h or more), where period difference between successive groups was on average 1.5 h. Their responses to lithium were determined experimentally.

The drug responses from control (Fig. S2B, C) and BD cells assigned to these categories exhibited a clear spectrum of dose-dependent responses (Fig. 2b, Fig. S2D). Significant differences were evident between dose and basal circadian period: long cells displayed lower period lengthening responses over 10–30 mM relative to short and medium cells (Fig. 2c). Lithium was found to be incapable of increasing the period beyond 27 h, regardless of the basal period. This attenuated period increase in patients with longer periods implies a plateau response in the case of lithium. Nevertheless, the altered lithium’s dependence on the basal circadian period (even when not approaching 27 h) suggests a defect in the mechanism underpinning the clock machinery. In contrast, amplitude enhancement was uniformly observed (Fig. 2d) across all patient subgroups and lithium concentrations (Fig. 2d), thus demonstrating clear differences in lithium’s effects on period and amplitude. A differential effect of lithium—affecting one property of the clock without affecting the other has not been described. This provides compelling support for the notion that these two properties are controlled independently [34]. Thus, as hypothesized, differences in lithium-induced period lengthening are determined by the underlying circadian phenotype.

To determine whether these differential chronomodulatory responses are unique to lithium or a general circadian response of long-phenotype patients, two additional compounds were assessed: nobiletin (NOB), a polymethoxylated flavone [41] and CGS-15943 (CGS), a nonspecific adenosine antagonist [42]. These function via different primary targets and pathways, but show lithium-like phenotypic effects on the clock.

NOB preferentially increased period in our short-period fibroblasts at 10–30 μM, by up to 1.5 h (Fig. 2e). All categories presented amplitude enhancement, much as for lithium. Whilst no effect for basal period was identified, the clear and identifiable trend was that of muted amplitude enhancement within long-period cell lines (Fig. 2f). Therefore, NOB displayed trends for reduced chronomodulatory effects in long-phenotype patient cells similar to lithium.

Unlike lithium, CGS induced period lengthening across all categories. Cells derived from patients within the short, medium, or long category displayed maximal period lengthening by up to 12.6, 11.3, and 10.9 h (Fig. 2g), consistent with the trend for long-phenotype displaying muted responses. This further demonstrates that the observed ceiling of lithium’s period increasing effect (~27 h) is specific for lithium (Fig. 2c) rather than a category-wide phenomenon, as seen with CGS (Fig. 2g–h).

Collectively, built on these observations we hypothesized that the lack of lithium’s effect in long-period cells was due to an inherent fault in the circadian network rather than a ceiling effect. Therefore a drug capable of shortening circadian period should demonstrate a similar divergent effect across patient groups. Indeed, this proved to be the case with 6-bromoindirubin-3′-oxime (BIO), a GSKβ inhibitor that has previously been shown to shorten circadian period (Fig. S2E, F) [34]. In a dose-response study conducted with BIO, the short-period cells responded as predicted, with period shortening. In contrast the long-period cells failed to demonstrate any shortening, in fact, they responded with a mild period increase. Thus, along with lithium, NOB, CGS, and BIO induced differential chronomodulatory responses conditional upon basal circadian rhythms.

### Fibroblasts’ lithium responses do not discriminate between lithium-treated and non-treated patients

**Fig. 3:**
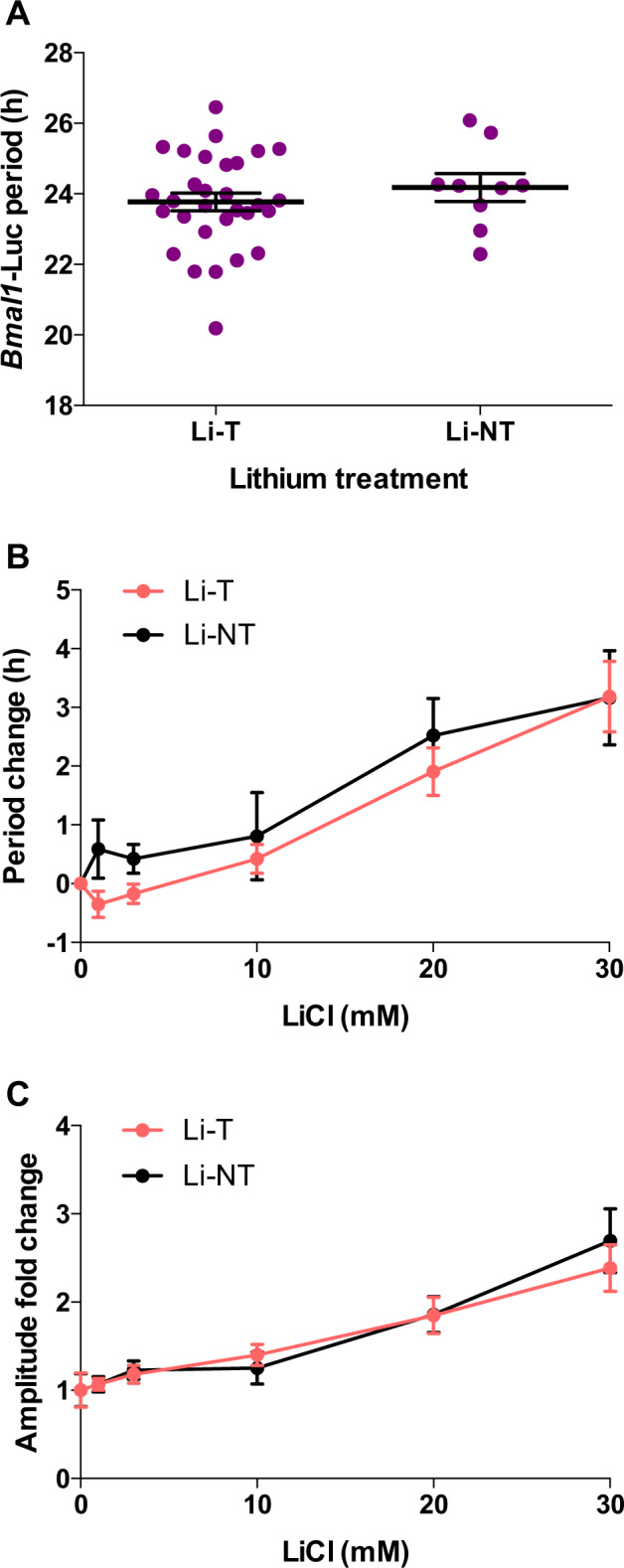
Fibroblast lithium response do not discriminate between lithium-treated and non-treated patients. **a** BD patients divided according to lithium treatment; basal period differences analyzed using a two-tailed Student’s *t* test. **b** Period and **c** amplitude effects induced by LiCl in fibroblasts from Li-T (*n* = 10) and Li-NT patients (*n* = 6), analyzed using a two-way ANOVA with a Sidak’s post hoc test. Data are presented as mean ± s.e.m.

As our BD subject-derived cells were shown to display patient-specific chronomodulatory responses to lithium, we were curious to understand whether cells from lithium non-medicated (Li-NT) patients would display any aberrant pharmacological responses. Basal circadian period showed no significant differences between the groups (Fig. 3a). However, patients not taking lithium are not lithium non responders, as patients can decline lithium for other reasons (e.g., side effects, preference, or disbelief in the diagnosis). Li-T and Li-NT cells displayed comparable period lengthening in response to lithium (Fig. 3b); both groups contained patients from the short, medium, and long categories. Similarly, Li-T and Li-NT cells exhibited non-differential dose-dependent amplitude enhancement (2.4-fold and 2.7-fold, respectively) with 30 mM LiCl (Fig. 3c).

### Nobiletin and CGS-15943 induce lithium-like behavioral changes in rodents

**Fig. 4:**
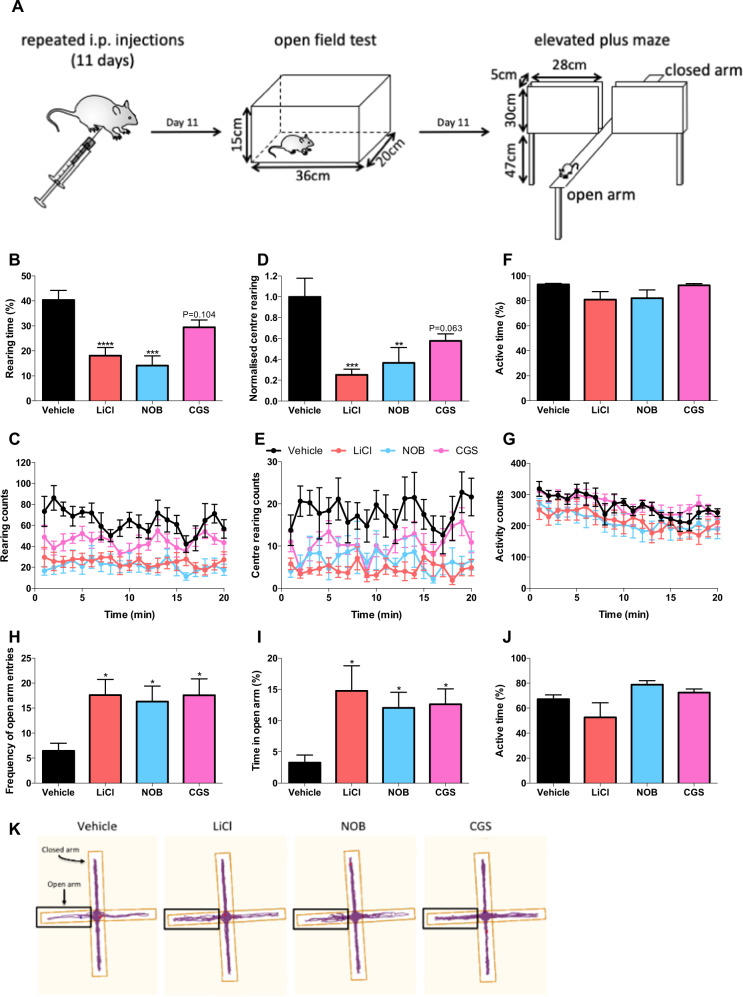
Nobiletin and CGS-15943 as pharmacological modulators of circadian rhythms mimic behavioral changes induced by lithium. **a** C57BL/6J mice were administered (i.p.) vehicle (*n* = 7), LiCl (*n* = 6), NOB (*n* = 7), or CGS (*n* = 7) then subjected to OFT and EPM behavioral tests. OFT: effects of compounds on (**b**, **c**) time spent rearing, (**d**, **e**) normalized number of center rears with representative traces, and (**f**, **g**) time spent active. EPM (1.5–2 h after the OFT): effects of compounds on (**h**) number of entries into the open arm, (**i**) time spent in the open arm, and (**j**) time spent active. **k** Representative tracking plots. Blue and red dots indicate mice positions when analysis began and ended, respectively. Data were analyzed using a one-way ANOVA with a Dunnett’s post hoc test and are presented as mean ± s.e.m. *p < 0.05; **p < 0.01; ***p < 0.001; ****p < 0.0001.

Both NOB and CGS displayed lithium-like chronomodulatory changes dependent on the underlying basal period (Fig. 2e–h). Importantly, the three compounds exert their effects on the clock through different targets. We therefore decided to assess whether these compounds induce similar behavioral modifications. If true, this would suggest that pharmacological modulation of circadian rhythm directly modulates affective behaviors, thus providing the basis for a novel treatment paradigm. To this end, we monitored two well-validated lithium-specific behavioral changes [43]. Following acclimatization, wild-type C57BL/6J mice were administered i.p. injections of LiCl, NOB, or CGS. After the final dose on day 12, behavior was assessed using the OFT and EPM behavioral methods (Fig. 4a).

Rearing activity was monitored using the OFT. Lithium is well characterized to reduce rearing, an action indicative of exploration and impulsivity [43]. We found that lithium and NOB reduced the time spent rearing by 55 and 65% respectively; nonsignificant (27%) reductions were observed with CGS (Fig. 4b, c). In addition, mice treated with lithium, NOB, and CGS reduced the number of central rearing episodes by 75%, 63%, and 42% respectively (Fig. 4d, e). The overall activity of animals confirmed that our findings were not due to increased movement (Fig. 4f, g). Therefore, it was concluded that NOB and CGS show strong indications of lithium-like rearing behavior.

As lithium also modulates anxiety-related behaviors, we tested behavioral responses on the EPM [43]. Mice treated with LiCl, NOB, or CGS were more likely to enter the open arms (2.7-, 2.5-, and 2.7-fold respectively; Fig. 4h), confirming that all three compounds possess anxiolytic-like effects. Furthermore, mice treated with LiCl spent 4.5-fold more time within the open arms; similarly, administering NOB or CGS increased the time in the open arms by 3.7- and 3.9-fold, respectively (Fig. 4i). Much like our findings with the OFT, these compounds did not affect the amount of time spent active (Fig. 4j). Therefore, as exemplified by the tracking plots (Fig. 4k), NOB, and CGS phenocopied lithium-induced behavioral modification.

### *Cry1/2*^*−/*−^ mice display atypical behavioral changes with lithium treatment

**Fig. 5:**
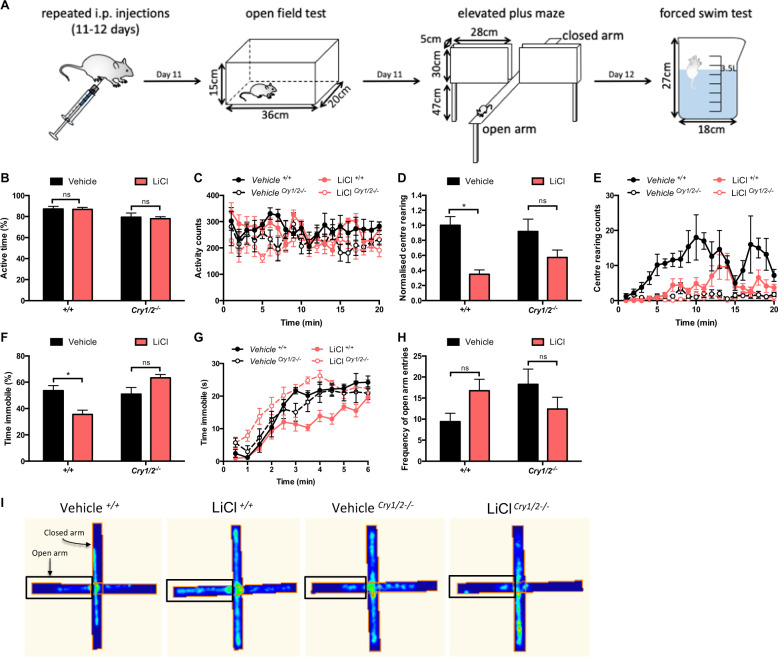
Lithium treatment differentially modulates behavior in *Cry1/2*^*−/−*^ mice. **a** Wild-type and *Cry1/2*^*−/−*^ mice were administered (i.p.) vehicle or LiCl (vehicle^*+/+*^: *n* = 6, LiCl^*+/+*^: *n* = 7, vehicle^*Cry1/2−/*−^: *n* = 8, LiCl^*Cry1/2−/−*^: *n* = 8) and tested by OFT, EPM, and FST. OFT effects of lithium on (**b**), (**d**) time spent active and the normalized number of center rears, with (**c**), (**e**) representative traces to visualize the treatment and genotype differences in response to lithium. FST effects on (**f**), (**g**) time spent immobile. **h** EPM number of entries into the open arm. **i** Heat maps from representative mice highlighting behavioral differences in the EPM. Data were analyzed using a two-way ANOVA with a Sidak’s post hoc test and presented as mean ± s.e.m. *p < 0.05.

To understand whether a dysfunctional clock is detrimental for lithium treatment, we performed behavioral tests using *Cry1/2*^−*/−*^ mice. These mice have known circadian deficits, including arrhythmicity, and reduced circadian amplitude under constant conditions [44]. Thus, if the molecular clock plays a part in mediating lithium’s effect, these should be altered in the *Cry1/2*^*−/*−^ mice. Following i.p. injections of LiCl, behavior was evaluated using the OFT, EPM, and FST (Fig. 5a).

There were no overall OFT differences in the in active time between lithium- and vehicle-treated animals (Fig. 5b, c). As expected, lithium reduced central rearing behavior in wild-type mice by 65%; this was muted in *Cry1/2*^−*/*−^ mice along with total time spent rearing (Fig. 5d, e, Fig. S3A, B). In the FST behavioral test (also known as a despair test), lithium is known to increase the amount of time the animal is active [43]. Wild-type mice treated with lithium displayed a 34% reduction in immobility, whereas no significant differences were observed for *Cry1/2*^*−/*−^ mice (Fig. 5f, g; Fig. S3C). In the EPM test, nonsignificant divergent trends for increased frequency of open-arm entry and time spent in the open arms were observed between these groups (Fig. 5h, i, Fig. S3D). Importantly, lithium-altered behavioral response has also been demonstrated with *ClockΔ19* mice [17]. Collectively, these data show that the typical lithium-induced behavioral changes are significantly altered in mice with dysfunctional clocks.

## Discussion

Here we show that patient fibroblasts exhibiting a long-period circadian phenotype exhibit atypical chronomodulatory responses to lithium. Crucially, we found that this aberrant response extends to a range of chronomodulators that act via different molecular pathways, thus suggesting a fundamental defect in the clock pathways. Further, using circadian mutant mice we show that a functional clock is required to exhibit typical lithium-mediated behavioral responses. These data provide evidence to suggest that patients with a dysfunctional circadian clock may not exhibit typical clinical responses to lithium therapy. Importantly, we also show that drugs that phenocopy lithium at the cellular level elicit behavioral responses akin to lithium in mice. Taken together, the results strongly encourage the exploration of chronomodulation as a novel route to achieving mood stability, with the potential to translate into a new treatment paradigm for BD.

We could not confirm previous reports of longer circadian cycles in fibroblasts from patients with BD using two independent reporters. However, patients that exhibit a wider distribution of circadian period lengths and longer basal periods are associated with greater polypharmacy, indicating possible lower effectiveness of lithium in these patients. This is supported by the observation that longer basal circadian periods are associated with muted responses to chronomodulatory interventions with lithium. If lithium differentially impacts the circadian system and its efficacy is dependent on the patients’ underlying circadian phenotype, this finding has important mechanistic and clinical implications. Pharmacological agents that modulated circadian rhythmicity in vitro also induced lithium-like behavioral changes in mice.

Using virally transduced *Bmal1*-Luc and *Per2*-Luc subject-derived cells, we were unable to identify any significant differences in basal period length. This is in contrast to a previous study, which identified significant *Per2* period lengthening in BD by 25 min [26]. However, in accordance with our investigation, no significant effects have been observed with *Bmal1-*Luc-transduced fibroblasts or mRNA analysis of patient cells [27, 45]. There are several reasons why we believe our data provide an accurate representation of the circadian state associated with BD. Firstly, the StemBANCC consortia rigorously selected patients to create a bio-bank for hard-to-treat disorders and thus fully phenotyped patients for subsequent studies. Secondly, the utilization of more patient cell lines (*n* = 39 in our study vs. *n* = 12–19 in previous studies) may account for the wider spectrum of periods associated with BD. Finally, the lack of *Bmal1* period lengthening in BD reported here is further strengthened by our observation showing a similar lack of period increase in BD using the *Per2*-reporter, further validating these findings. Whilst it would be useful to compare basal circadian amplitude between different patient cell lines due to previous reports of BD patients demonstrating muted amplitude, the number of viral integrations per fibroblast dictates the observed basal amplitude in our circadian assays. Thus, amplitude could simply be higher if there were more copies of the *Per2* or *Bmal1* gene inserted into the genome. However, basal period and drug-induced period and amplitude changes are independent of the integration factor and were therefore our primary parameters.

The clinical features of the patients did not readily explain the period variability observed within our BD cohort. Whilst our control cohort had a balanced sex distribution (F:M, 55:45), the BD cohort had a female bias (F:M, 74:26). Furthermore, the control cohort was on average 10 years older (BD 43 years vs. control 53 years). However, given there was no correlation between age and sex with basal circadian period or lithium response (Fig. S1) these factors are unlikely to account for our observed differences. Further, to take account of the variable sample numbers between the control and BD cohort, we used Welch’s *t* test which is better suited to handle variable sample sizes and variances [46, 47]. Disease diagnosis showed that BP-II patients had a shorter period than BP-I patients (by 1.1 h, *p* = 0.054). Although there are no reports directly comparing cellular rhythmicity in BP-I vs. BP-II patients, differences in sleep have been identified, and sleep efficiency and duration were improved only in BP-I patients taking lithium [48]. However, due to the limited sample size, it would be prudent to replicate this study with a larger cohort; if the observation is validated, this could enable the identification of appropriate chronomodulatory compounds in the future.

Our investigation confirms previous findings that lithium induces period lengthening and increases amplitude [28, 34, 49, 50]. Further, we report that patient cells with longer circadian periods show reduced circadian responses to lithium. We also demonstrate that this deficit applies to other chronomodulatory compounds: CGS, NOB, and BIO (both period increasing and decreasing modulators). As CGS, NOB, and lithium function via distinct primary targets and cellular mechanisms (CGS: nonspecific adenosine antagonist [42]; NOB: multiple targets, including retinoid orphan receptor [41] and CREB [51]; lithium: multiple targets e.g., IMPase, GSK3β [52]), such altered chronomodulatory effects in patient fibroblasts in response to these drugs are unlikely to be mediated by changes in activity associated with a single target. Rather, our observations point to a general mechanistic aberration in these patients’ clocks and the chronomodulatory agents driving the clock to an altered equilibrium to overcome these functional deficits. Indeed, the specific observation that lithium exerts differential circadian effects is supported by a recent study [53]. It is unlikely that the longer phenotype is a consequence of long-term medication, as the cells were passaged at least 4–6 times since biopsy, during which time the drugs would have washed out, excluding potential epigenetic changes. Further, there was no difference in circadian responses between Li-T and untreated cells. Whilst we cannot conclude that control-subject derived fibroblasts would not also show similar effects, such subjects would never need to take lithium therapeutically. Therefore, our primary focus was on understanding the lithium response across BD patients. Overall, we show that drug responses vary in BD patients based on the underlying cellular circadian rhythms. Thus, currently unidentified factors that are implicated in the functioning of the molecular clock may be significantly disrupted in patients with longer basal circadian periods. If this can be validated on a larger scale, with mood regulation as an additional outcome, patient-derived fibroblasts could become a meaningful biomarker and facilitate the implementation of a personalized approach to lithium treatment. Whilst lithium’s established effects require long-term therapy to become apparent, we show almost immediate changes to circadian rhythms in our cell model. It has been shown with several other mood modulators that even in the absence of measurable changes in subjective mood, changes in emotional processing and memory are apparent after a single treatment [54]. This suggests that whilst the neurophysiological and mechanistic changes induced by these therapeutics are almost immediate, their translation into improved symptoms exhibit a lag [55]. Similarly to these agents, we hypothesize that beneficial alterations to circadian rhythms will initiate neurophysiological changes and promote improvements in patient symptoms.

Although screening for novel clock modulators has been performed on immortalized cells [56–58], as we do not fully understand BD, use of our patient-derived fibroblasts have several advantages over existing models. Fibroblasts provide a more biologically relevant representation of the individual’s genetic makeup, and they represent a valid indication of human physiological circadian rhythmicity (Table 1). This therefore provides researchers with a more accurate model of the disease, which may help identify patient-relevant pharmacological treatments in the future. Furthermore, the patients from whom our fibroblasts originated were extensively evaluated: each cell-line was accompanied by a wealth of long-term phenotypic information. Therefore, all these 39 fibroblast lines could be utilized in the future, or particular cell lines could be selected based on the research needs or the genetics behind the differential drug responses be explored. The creation of a patient-derived fibroblast model also significantly reduces the need for animals, including transgenic mice to model circadian rhythm deficits in mood disorders; replacing with a human relevant model will be highly beneficial at various stages of drug discovery, including high-throughput screening.

This investigation also found that the novel chronomodulatory drugs NOB and CGS reduce rearing and anxiety-related behavior, similar to lithium. This supports the theory that drugs that modulate circadian rhythm can alter temperament. Indeed, several lines of study support this; key examples include the fact that the melatonergic agonist agomelatine and light therapy normalize extreme behavioral irregularities such as depression, mania, and delusions [59–62]. Furthermore, work in *ClockΔ19* mice has shown that lithium-induced behavioral responses are altered in a range of behavioral tests including EPM, OFT, and FST, thus yielding evidence for interactions between lithium, the molecular clock, and behavior. To provide further evidence that these effects are not specific to the *ClockΔ19* genotype, we performed comparable behavioral experiments and showed that lithium’s effects are indeed altered in *Cry1/2*^−*/−*^ mice, compared with controls. Collectively, these investigations provide evidence for the involvement of the molecular clock in lithium’s behavioral effects.

In conclusion, patient-derived fibroblasts enabled the identification of differential drug responses based on basal circadian rhythms. This advances our understanding of the role of circadian rhythms in BD and supports the wealth of evidence demonstrating a close relationship between circadian rhythms and the pathophysiology of BD. If pharmacological modulation of circadian rhythms can provide a novel method to achieve mood stability, it will be necessary to validate these findings with additional molecules and in larger studies in the hope of identifying a new treatment paradigm for BD and perhaps other neuropsychiatric disorders.

## Supplementary information

Supplemental material
