# Supplementary material for: Patient fibroblast circadian rhythms predict lithium sensitivity in bipolar disorder
Source: Mol Psychiatry. 2020 May 13;26(9):5252–65. doi: 10.1038/s41380-020-0769-6 (PMC8589670; doi:10.1038/s41380-020-0769-6)
Supplement: Supplementary file 1 — Supplemental material [file 41380_2020_769_MOESM1_ESM.docx]

**Supplementary Figure 1**

**Supplementary Fig. 1 Bipolar patients exhibit a wider range of period distributions relative to healthy controls.** **A)** *Per2*-Luc periods assessed in a subset of control (n=12) or BD patient (n=18) fibroblasts. **B)** Exemplar oscillations obtained from control and BD patient cell line transduced with the *Per2*-Luc lentivirus. **C)** The overall population doubling time for control (n=23) and BD (n=39) cells. Stratification of BD patients into categories with their corresponding periods, including division by: presence of psychotic symptoms **(D),** sex **(E, G)**, and age **(F, H)**. Sex and psychosis were analyzed using a Student’s t-test and age was analyzed using a one-way ANOVA with a Tukey’s post-hoc test. Data presented as mean ± s.e.m.

*Growth Curves for estimating population doubling times*

Human fibroblasts were grown and seeded at 15,000 cells/well in 6-well plates (Corning). The cells were trypsinised in triplicates and counted on days 1, 4, and 8 to assess the population doubling time. A selection of cell lines were trypsinised and counted every day to confirm the pattern represented was indicative of standard growth curves. Cell counting was performed using the Countess Automated Cell Counter (ThermoScientific) and population doubling time was calculated using the formula below. $\mathcal{T}$: incubation time in days, $\mathcal{Xb}$: cell number on day 1, $\mathcal{Xe}$: cell number on day 8.

$$population doubling time=\mathcal{T}\frac{\mathcal{ln}2}{\mathcal{ln(Xe \div Xb)}}$$

**Supplementary Figure 2**

**Supplementary Fig. 2 Lithium induces chronomodulatory changes in cells derived from both patient and healthy controls. A)** Exemplar traces from patient-derived cells belonging to the short (period=23.49h or less), medium (period=23.50-24.99h), or long (period=25.00h or more) categories. The period **(B)** and amplitude **(C)** effects induced by 1-30mM LiCl in control subject-derived fibroblasts (n=8). Data was analyzed using a one-way ANOVA with a Dunnett’s post-hoc test. **D)** Illustrative traces highlighting the lithium-induced chronomodulatory changes in cell lines from the short, medium, or long groups. **E-F)** The circadian period effects of 6-bromoindirubin-3'-oxime on BD patient fibroblasts with short (n=5) or long (n=5) basal period lengths with representative traces. Data presented as mean ± s.e.m. *p<0.05, ****p<0.0001.

**Supplementary Figure 3**

******

**Supplementary Fig. 3 Additional behavioral differences observed between wild-type and *Cry1/2^-/-^* mice with lithium treatment. A-B)** The differences in genotype associated with vehicle or LiCl treatment (vehicle*^+/+^*: n=6, LiCl*^+/+^*: n=7, vehicle*^Cry1/2-/-^*: n=9, LiCl *^Cry1/2-/-^*: n=8) when observing rearing in the OFT. Genotype effects for the time spent mobile in the FST **(C)** and the amount of time spent in the open arms of the EPM **(D)**. The data were analyzed using a two-way ANOVA with a Sidak’s post-hoc test. Data presented as mean ± s.e.m. *p<0.05.

**Supplementary *Table 1* Demographic summary of all the subjects used in the current investigation.**

|  |  | |  | | Sex: | | | Diagnosis: | | Age of first impairment: (years) | | |  |
| --- | --- | --- | --- | --- | --- | --- | --- | --- | --- | --- | --- | --- | --- |
| Subject | | **n** | | **Age (years)** | | **Female** | **Male** | **BP-I** | **BP-II** | **Depression** | **Mania** | **Psychotic symptoms** | |
| All BD | | 39 | | 43.10 ±1.97 | | 74% | 26% | 82% | 18% | 18.74±1.30 | 23.03±1.57 | 67% | |
| Li-T | | 30 | | 43.03±2.39 | | 70% | 30% | 83% | 17% | 19.83±1.56 | 23.10±1.72 | 67% | |
| Li-NT | | 9 | | 43.33±3.94 | | 89% | 11% | 78% | 22% | 15.11±1.84 | 22.78±3.84 | 67% | |
| Control | | 23 | | 53.56±3.18 | | 55% | 45% |  |  |  |  |  | |

**Supplementary *Table 2* The features associated with lithium-treated patients.**

|  |  | |  | |  | Lithium prevents mood episodes: | | | >2 years compliance: | |  | Lithium monotherapy: | |
| --- | --- | --- | --- | --- | --- | --- | --- | --- | --- | --- | --- | --- | --- |
| Subject | **Age (years)** | **Currently on lithium** | | **Consistent time on lithium (years)** | | | **Yes** | **Not sure** | **Yes** | **No** | **Effectiveness of lithium**  **(1 to 10)** | **Yes** | **No** |
| Li-T | 43.03±2.39 | 100% | | 10.19±0.24 | | | 80% | 20% | 97% | 3% | 6.00±0.49 | 20% | 80% |

**Supplementary *Table 3* The medications taken by patients were categorized by indication.**

| Class | Medications |
| --- | --- |
| Mood stabilizers | lithium, lamotrigine, valproate |
| Antipsychotics | aripiprazole, chlorpromazine, olanzapine, quetiapine, risperidone |
| Antidepressants | amitriptyline, citalopram, escitalopram, mirtazapine, sertraline |
| Anxiolytics | diazepam, lorazepam |
| Hypnotics | zolpidem, zopiclone |

**Supplementary *Table 4* Summary of the total and average bipolar patient pharmacological treatments.**

| Subject | Total number of drugs | Mood stabilizers | Antipsychotics | Antidepressants | Anxiolytics | Hypnotics |
| --- | --- | --- | --- | --- | --- | --- |
| All BD | **92**  2.35±0.21 | **54%**  1.28±0.11 | **25%**  0.59±0.10 | **9%**  0.21±0.08 | **7%**  0.15±0.04 | **5%**  0.13±0.05 |
| Li-T | **76**  2.53±0.24 | **59%**  1.50±0.10 | **24%**  0.60±0.11 | **8%**  0.20±0.09 | **5%**  0.13±0.06 | **4%**  0.10±0.06 |
| Li-NT | **16**  1.78±0.36 | **31%**  0.56±0.18 | **31%**  0.56±0.18 | **13%**  0.22±0.15 | **13%**  0.22±0.15 | **13%**  0.22±0.15 |
